# Conversion therapy and the LGBT community: the role of the College now?

**DOI:** 10.1192/bjb.2018.90

**Published:** 2018-12

**Authors:** Annie Bartlett

**Affiliations:** Professor of Offender Healthcare, St. George's, University of London, UK; email: abartlet@sgul.ac.uk

The history of the relationship between the LGBT (lesbian, gay, bisexual and transgender) community and psychiatry has not been a happy one, reflecting but also at times reinforcing hostile social attitudes. More recently, both psychiatry and psychological therapies have been at pains to distance themselves from previous discriminatory practice and align themselves with more contemporary understandings of diverse sexual identities and behaviour. The College and other bodies such as the UK Council for Psychotherapy have developed clear statements on sexual orientation and the issue of conversion therapy in particular, pointing out that it does not work, can create distress and should not be undertaken.^1^

This position on conversion therapy is echoed in the raft of 75 measures announced recently by Women and Equalities Minister Penny Mordant, based on the results of the largest survey of LGBT people ever undertaken. The measures are designed to create a more inclusive society in which, among other things, individuals of any sexual persuasion may feel able to hold hands in public without fear of ridicule or attack (Hansard HC Deb, 3 July 2018, cWS). That a Conservative government has launched such an initiative seems remarkable to those of us able to remember Section 28, brought in by the Thatcher government 30 years ago (Local Government Act 1988).

However, we should be mindful as a profession that some practitioners may not be so sympathetic to this direction of travel. Not so long ago, a significant proportion of psychiatrists and therapists, 4%, were still prepared to treat individuals for their gay and lesbian identities.^2^ It is not clear how much this has changed, if at all, on the ground. Equally, it is still very easy to locate reparative therapy options on the internet, a common port of call for those wanting some help negotiating same sex sexual preferences. A quick search picks up, among others, Nicolosi's website, which continues to say ‘If gay doesn't define you you don't have to be gay,’ accompanied by claims of treatment efficacy, in line with the views he so publicly expressed until his death in 2017 (https://www.josephnicolosi.com). He recommended reparative therapy, saying that it worked and should be offered as a psychological therapy. The number of individuals currently involved with reparative therapy is unclear and seems likely to be difficult to establish, as recent medical and political pronouncements may well drive it underground.

The College has an international reach. Many countries are still much more punitive to LGBT individuals than the UK; some have the death penalty on their statute books. Major world religions are internally divided on LGBT issues and can legitimise not only adverse social attitudes but also attempts at religious rather than medical cures. It remains to be seen how energetic the College feels it should be in sharing its liberal understanding of sexual preference. Its position on change-oriented treatments of any kind has international applicability. There may well be many individuals overseas who would thank psychiatry for taking part in this ongoing debate.
